# Microscopic and Macroscopic Fragmentation Characteristics under Hypervelocity Impact Based on MD and SPH Method

**DOI:** 10.3390/nano11112953

**Published:** 2021-11-04

**Authors:** Wei-Dong Wu, Jin-Ming Liu, Wei Xie, Yan Xing, Jian-Li Shao

**Affiliations:** 1 State Key Laboratory of Explosion Science and Technology, Beijing Institute of Technology, Beijing 100081, China; wu_weidong93@163.com; 2 Defense Engineering Institute Academy of Military Sciences, Beijing 100039, China; Liujm1025@outlook.com (J.-M.L.); xieweixiongqi@163.com (W.X.); xingyan131@163.com (Y.X.); 3 Explosion Protection and Emergency Disposal Technology Engineering Research Center of the Ministry of Education, Beijing 100081, China

**Keywords:** fragmentation, molecular dynamics, Smoothed Particle Hydrodynamics, aluminum

## Abstract

This work investigates the difference in the fragmentation characteristics between the microscopic and macroscopic scales under hypervelocity impact, with the simulations of Molecular Dynamics (MD) and Smoothed Particle Hydrodynamics (SPH) method. Under low shock intensity, the model at microscopic scale exhibits good penetration resistance due to the constraint of strength and surface tension. The bullet is finally embedded into the target, rather than forming a typical debris cloud at macroscopic scale. Under high shock intensity, the occurrence of unloading melting of the sample reduces the strength of the material. The material at the microscopic scale has also been completely penetrated. However, the width of the ejecta veil and external bubble of the debris cloud are narrower. In addition, the residual velocity of bullet, crater diameter and expansion angle of the debris cloud at microscopic scale are all smaller than those at macroscopic scale, especially for low-velocity conditions. The difference can be as much as two times. These characteristics indicate that the degree of conversion of kinetic energy to internal energy at the microscopic scale is much higher than that of the macroscopic results. Furthermore, the MD simulation method can further provide details of the physical characteristics at the micro-scale. As the shock intensity increases, the local melting phenomenon becomes more pronounced, accompanied by a decrease in dislocation atoms and a corresponding increase in disordered atoms. In addition, the fraction of disordered atoms is found to increase exponentially with the increasing incident kinetic energy.

## 1. Introduction

The hypervelocity impact of projectiles on thin plates is mainly studied to optimize spacecraft shields and evaluate projectile penetration capabilities in defense applications [1]. After penetrating the thin plate, there will form many small fragments, and the debris cloud will be generated. Therefore, it shows great significance in protecting the spacecraft to investigate the size, velocity and distribution of debris.

Experimental research, theoretical analysis and numerical simulation are the technical basis for studying hypervelocity impact. Considerable experimental research [2,3,4,5,6,7] and theoretical analysis [8,9,10,11,12,13] has been carried out over the past few decades. Many aspects, such as the formation process, distribution characteristics, theoretical models and penetration performance, have been investigated in-depth in understanding the dynamics responses of a debris cloud. The ground experiment is the most direct method of hypervelocity impact research, but the test is limited by launch capability and diagnostic equipment. It is very difficult to obtain the detailed process of the debris formation under the extreme conditions. Therefore, the numerical simulation technology has become an auxiliary tool for many researchers to study the debris cloud characteristics. Commonly used methods include the Lagrange, Euler, ALE and Smooth Particle Hydrodynamic (SPH) methods. As for calculating the large deformation problem, the mesh deformation of the Lagrange method is very serious, which eventually leads to the failure of the calculation [14,15,16]. The Euler method is difficult to track the position of the interface and has the disadvantages of long calculation time and poor calculation accuracy [17,18]. The SPH method is the most effective method to simulate the phenomenon of large deformation and high strain rate in hypervelocity impact. The evolution history of SPH in detail was reported by Liu et al. [19]. Based on the SPH and Euler methods, Fahrenthold et al. [20] studied the effectiveness of the Grady-Kipp fragmentation model for debris cloud characteristics. Silnikov et al. [21] used the SPH method to compare the debris cloud characteristics of spherical, cylindrical and cubic projectiles impacting a thin plate. Their results show that in case of the cube sharp edge impact, a debris cloud of a higher density is formed. Verma and Dhote [1] simulated the impact of stainless steel spherical projectile against a mild steel shield. They obtained the constants and radicals for the empirical equations using the simulation data. It is worth noting that Chen et al. [22,23] recently proposed a FEM-SPH adaptive method, which converts the failed elements into SPH particles. The debris cloud characteristics obtained by this method are in good agreement with the experiment.

The dynamic damage of materials under extreme conditions is often accompanied by complex deformation, heating, melting, vaporization and even phase change [24]. Therefore, it is very difficult to consider all influencing factors at the same time when constructing a theoretical damage constitutive model. Correspondingly, numerical simulation methods of different physical scales have been developed to investigate the dynamic properties of materials [25,26,27,28,29]. In particular, molecular dynamics (MD) simulation has attracted more and more attention recently [30,31,32,33,34]. As early as 1987, Holian et al. [35] studied the hypervelocity impact of a spherical projectile against the thin plate based on molecular dynamics. There are striking similarities and significant differences in the debris cloud phenomenon between the microscopic scale and the continuum hydrodynamics simulations. Steinhauser et al. [36] used the discrete spherical particles interacting with potential functions to build a solid. They found that using this particle simulation method leads to very stable, energy conserving simulations of hypervelocity impact that correspond to experiments. On the whole, due to the strong surface and crystal orientation effects, the dynamic responses of nanomaterials will show more diverse phenomena. A large number of studies have found that the mechanical and physical properties of the material will show different laws from the macroscopic conditions when the material size is reduced to a certain extent, usually called the size effect [37]. The size effect may show a variety of characteristics under different materials and dynamic environments.

In this work, we analyze the fracture process and result characteristics by simulating the same impact speed and model at different scales, and further understand the difference of failure characteristics between the nano-scale and the macro-scale materials. Based on the SPH and MD methods, the laws of fragmentation characteristics, including the debris cloud evolution, stress distribution, residual velocity, crater diameter and expansion angle, are compared. The structure of this article is as follows. The detailed information of the methods and simulation details are described in Section 2 and Section 3, respectively. The results and discussion are demonstrated in Section 4. The conclusion is summarized in Section 5.

## 2. Numerical Simulation Method

### 2.1. Molecular Dynamics

Molecular Dynamics (MD), first developed in the late 1970s, is a method of obtaining material property information by solving the motion equation of each particle based on the interaction between particles. The molecular dynamics simulation method is based on Newton’s second law. The potential function U is used to describe the force of all other particles in the system acting on the investigated particle. Therefore, for a system containing *N* particles, the force exerted on the particle *i* can be obtained

(1) $$\{\begin{array}{c} F_{i}=m_{i}a_{i}=-\frac{\partial U}{\partial r_{i}}\\ v_{i}\left(t\right)=\frac{\partial r_{i}}{\partial t} \end{array}$$

where ***F***_i_ is the force exerted on the particle *i*. *m*_i_, ***a***_i_ and ***v***_i_ are the mass, acceleration and velocity of the particle *i*, respectively. The most common potential functions in metals are pair potential and embedded atom method. In order to solve the problem of complicated calculation and time-consuming computer time, the method of introducing the cutoff distance, rc, is usually adopted. When the distance between the other particles and the investigated particle exceeds rc, the inter-force is not calculated. Then, according to the coordinates and velocity of each particle, statistical methods are used to calculate the value of macroscopic physical quantities, such as stress **σ** and temperature *T*

(2) $$\sigma =\frac{1}{V}\overset{N}{\underset{i}{\sum }}\left[\frac{1}{2}\underset{j\neq i}{\sum }F_{ij}⊗r_{ij}+m_{i}v_{i}⊗v_{i}\right]$$

(3) $$T=\frac{1}{3Nk_{B}}\overset{N}{\underset{i=1}{\sum }}mv_{i}{}^{2}\;$$

where *V* is the atom volume, ***F****_ij_* is the force on atom *i* due to atom *j* and ***r****_ij_* is the position vector of atom *j* relative to atom *i*.

### 2.2. Smoothed Particle Hydrodynamics

As a kind of meshless method, Smoothed Particle Hydrodynamics (SPH) is used to avoid the limitations of mesh tangling encountered in extreme deformation problems with the finite element method. The SPH equation is constructed by the kernel interpolation and the particle approximation. The particle approximation of a function is as follows

**Figure 1:**
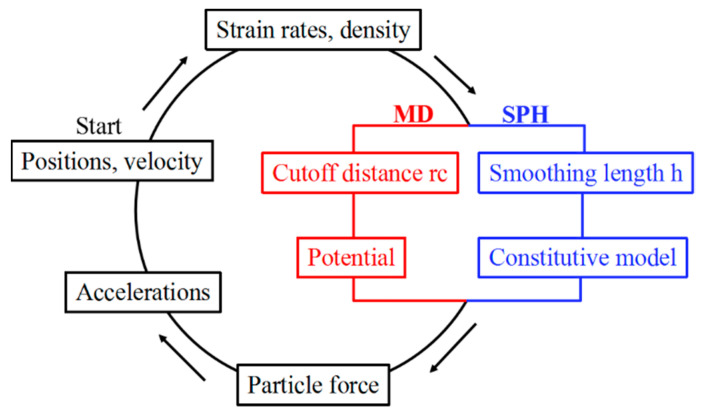
Comparison of calculation procedure between the MD and SPH methods.

(4) ∏f(x)=∫f(y)W(x−y,h)dy 

where *W* is the kernel function and *h* is the smoothing length. The kernel function is defined by the function *θ*, by the relation

(5) $$W\left(x,h\right)=\frac{1}{h{\left(x\right)}^{d}}\theta \left(x\right)\;$$

where *d* is the number of space dimensions. The most common smoothing kernel is the cubic B-spline, which is defined by choosing *θ* as

(6) $$\theta \left(u\right)=C\times \{\begin{array}{c} 1-1.5u^{2}+0.75u^{3}\\ 0.25{\left(2-u\right)}^{3}\\ 0 \end{array}\;\begin{array}{c} \left|u\right|\leq 1\\ 1<\left|u\right|\leq 2\\ \left|u\right|>2 \end{array}\;$$

where *C* is the normalization constant, which depends on the number of space dimensions. The particle approximation of a function can now be defined as

(7) $$\prod f\left(x_{i}\right)=\overset{N}{\underset{j=1}{\sum }}w_{j}f\left(x_{i}\right)W\left(x_{i}-x_{j},h\right)\;$$

where *w*_j_ = *m*_j_/*ρ*_j_ is the weight of the particle. According to the smooth kernel function, the cumulative function of the density, pressure and velocity of the investigated particle is obtained, and then the acceleration is derived, thereby simulating the movement trend of the system [19]. The diagram of calculating procedure for MD and SPH is displayed in Figure 1.

## 3. Simulation Details

**Figure 2:**
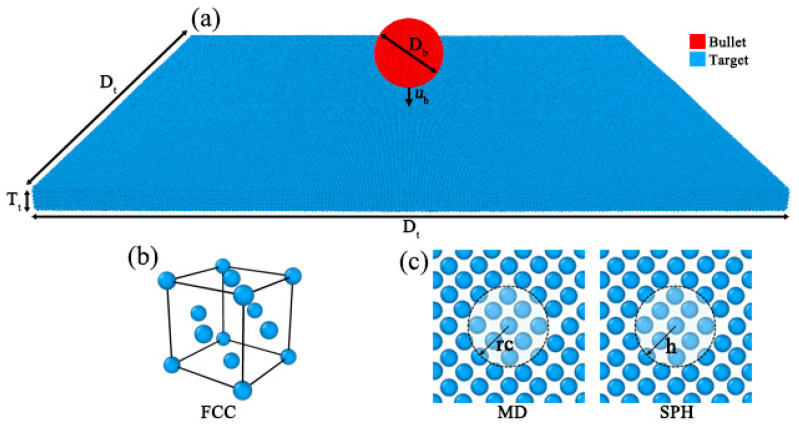
Particle configuration of MD and SPH. (**a**) The spherical bullet with diameter of *D*_b_ is located at the center of target. The total particle number and bullet-target size ratio between MD and SPH models are the same. (**b**) Both methods are face-centered cubic (FCC) structure. (**c**) The cut-off radius (rc) and the smooth core radius (h) represent maximum range of inter-particle force in MD and SPH, respectively.

The MD and SPH models (Figure 2) of the impact test are displayed. The simulation model consists of a square target part and a spherical bullet part. Both the target material and the bullet material are aluminum.

### 3.1. MD Computational Details

First of all, the target of 200 (*x*) × 200 (*y*) × 6.1621 (*z*) fcc unit cells is constructed. *x*, *y*, and *z* axes are, respectively, along the [100], [010] and [001] crystallographic directions. The lattice constant is taken as 4.05707 Å. The spherical bullet with the diameter of 10 nm is considered here. The embedded-atom method (EAM) potential developed by Zhakhovskii et al. [38] is adopted for Al, whose validity under strong shock conditions has been confirmed in previous work [30,31,32,33,34].

The above models are relaxed by energy minimization using the conjugate gradient method, followed by equilibration using the NVT ensemble at 300 K for 20 ps. After relaxation, the shock processes are simulated in micro-canonical ensemble (NVE), and free boundaries are set in all the three directions. The shock velocity up varies from 2 km/s to 10 km/s, which is added on the bullet along the z direction. All simulations are performed by open source LAMMPS code [39]. The visualization is done using OVITO program [40]. The temporal-spatial distributions of statistical physical properties are obtained by the method of dividing the cell into many bins, and then calculating the average value such as temperature, stress and velocity within each bin. The temperature is calculated by using the average kinetic energy in each bin after subtracting the contribution from the motion of the center of mass. The atomic stress is calculated according to virial formula. To identify the atomic level defects, the dislocation extraction algorithm (DXA) has been used.

### 3.2. SPH Computational Details

In order to ensure that the particle arrangement, the particle number and the bullet-target ratio are consistent with the MD model, we extract the coordinate information of all MD particles and then convert into SPH particles under the unit system of kg-s-m. The SPH model of the thermal-mechanical coupling analysis of hypervelocity impact is established using LS-DYNA software. The material type Mat_Johnson_Cook is applied to describe the mechanical behavior of Al under conditions of large deformation, high strain rate and high temperature. The equation of state type is Eos_Gruneisen. It is generally believed that most of the work done by plastic deformation will be converted into heat, which will cause a sharp temperature rise in the contact area of the bullet and target. The thermodynamic material type is Mat_Thermal_Isotropic. Here, the initial temperature of the whole model is 300 K. The constitutive parameters of the model [41] are shown in Table 1.

## 4. Results and Discussion

### 4.1. Analysis of Debris Formation Process

**Figure 3:**
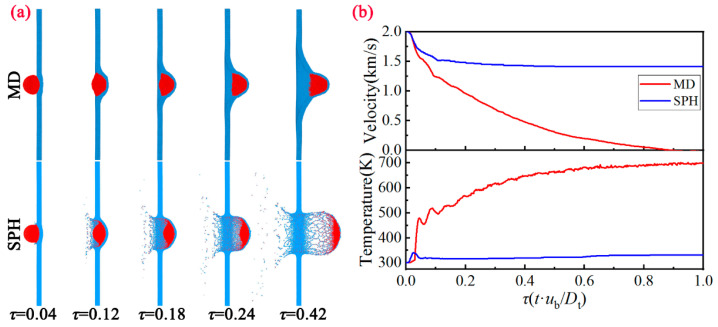
(**a**) Evolution of the debris cloud morphology between MD and SPH at different moments under *u*_b_ = 2.0 km/s. (**b**) Comparison of the velocity and temperature variation with time between MD and SPH.

To begin with, we compare the formation of the debris cloud at *u*_b_ = 2 km/s based on the MD method and SPH method, as shown in Figure 3a. Here, we make the time t dimensionless, and select the images corresponding to the same moment *τ* (=*t*·*u*_b_/*D*_t_) for comparison. It can be seen that for the MD method, the front-side of the bullet is severely deformed, and extends reversely along the impact contact surface. The impacted area of the target gradually dents inward, and eventually protrudes on the back of the target. Note that the bullet does not undergo disintegration, and the target is not perforated and destroyed, showing good anti-penetration characteristics. Meanwhile, for the SPH method, the bullet gradually disintegrates and the target is broken in the impacted area. Subsequently, the typical debris cloud is formed. As we all know, the debris cloud is usually composed of three parts: ejecta veil, external bubble and internal structure [6]. We can see that the ejecta veil is mainly formed by the reverse spraying of the material on the collision surface of the target. The external bubble is composed of the material on the back of the target, and the internal structure is composed of projectile fragments and the front-side of the external bubble.

Comparison of the velocity and temperature variation with time between MD and SPH is presented in Figure 3b. For the velocity variation of the bullet, the time history curve calculated based on the MD method continues to decay and finally drops to 0; while the SPH method has a significantly smaller attenuation degree and gradually enters a flat slope, and the final velocity is approximately stable at 1.5 km/s. For the temperature variation of the bullet, the MD method continues to rise and finally stabilize at about 700 K, while the SPH method rises rapidly to about 350 K during the impact process. As the debris cloud formed, the temperature drops gradually. These characteristics further show that the model exhibits good anti-penetration characteristics on the microscopic scale due to the constraint of strength and surface tension. Under this circumstance, the target completely absorbs the kinetic energy of the bullet, and transforms it into internal energy.

**Figure 4:**
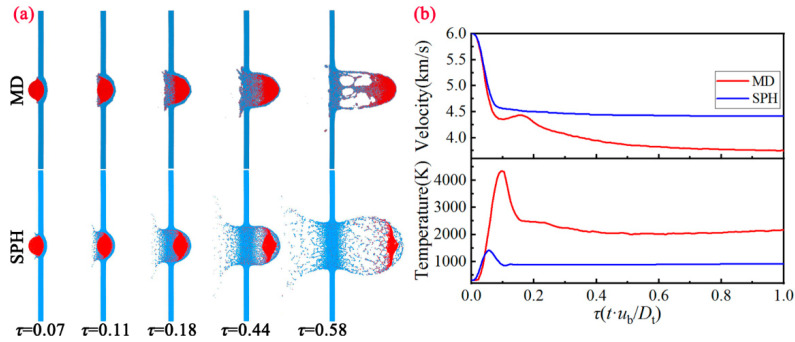
(**a**) Evolution of the debris cloud morphology between MD and SPH at different moments under *u*_b_ = 6.0 km/s. (**b**) Comparison of the velocity and temperature variation with time between MD and SPH.

Compared to the *u*_b_ = 2 km/s, the evolution of the debris cloud morphology at *u*_b_ = 6 km/s based on the MD method and SPH method is very different (Figure 4a). Under this shock intensity, both the MD method and SPH method will form the debris cloud. Furthermore, it can be seen that the front-side of internal structure is mainly composed of the bullet and target fragments, the center region is composed of bullet fragments, and the rear region is the semi-circular spalled fragment shells formed by spalling on the surface of the bullet. Compared with the typical debris cloud characteristics at the macro-scale (SPH), the debris cloud obtained based on the MD method shows distinct differences. In the region of ejecta veil and external bubble, both the lateral width and longitudinal length are narrower. More interestingly, the number of voids formed in the internal structure region is smaller, but the size is larger. The formation process of the debris cloud behaves more like a hydrodynamic process, showing obvious melting characteristics.

Comparison of the velocity and temperature variation with time between MD and SPH is presented in Figure 4b. For the velocity variation of the bullet, both the MD method and SPH method undergo the rapid deceleration stage, and gradually enter a flat slope. Note that the final velocity calculated by MD method is smaller than that by SPH method. For the temperature variation of the bullet, both the MD method and SPH method continue to rise. After reaching the maximum value (4500 K for MD, 1500 K for SPH), the temperature is reduced to approximately 2200 K (MD) and 900 K (SPH). Obviously, unloading melting has occurred in the sample under the strong impact intensity, thereby the strength of the material has been greatly reduced under this situation. It can be seen that the material at the microscopic scale has also been completely penetrated, but the calculation results are still significantly different from the macroscopic scale.

**Figure 5:**
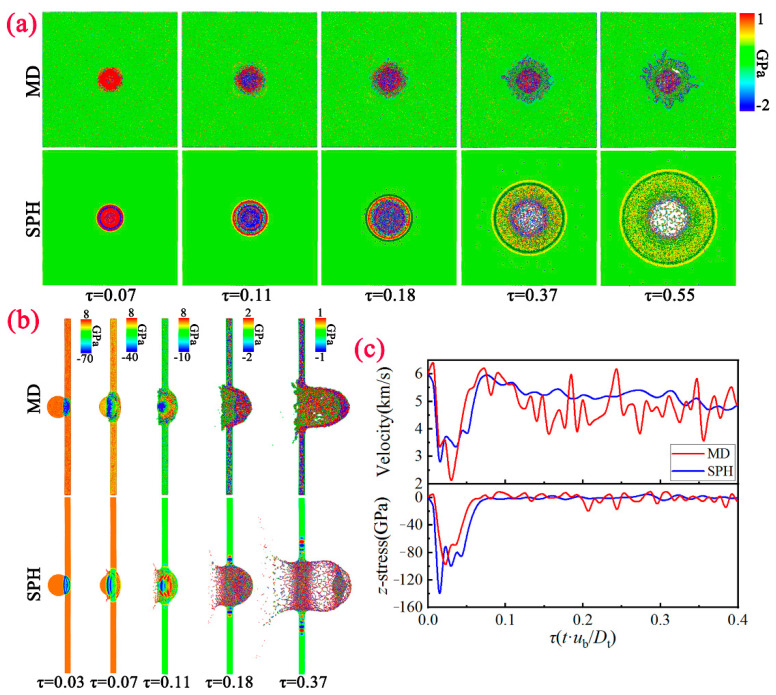
Views of the target pressure distribution (**a**) and the whole model z-stress distribution (**b**) between MD and SPH at different moments under *u*_b_ = 6.0 km/s. (**c**) Comparison of the velocity and z-stress variation with time of feature particle between MD and SPH.

To compare the propagation characteristics of the stress wave under these two methods, we first present the views of the target pressure distribution between MD and SPH at different moments in Figure 5a. Here, we take *u*_b_ = 6.0 km/s into account. It can be seen that the pressure gradually decreases as the damage degree of the target increases. More importantly, compared with the SPH method, the positive pressure value calculated based on the MD method is larger, while the negative pressure value is smaller due to the different characteristics of the debris cloud under these two methods. Moreover, it can be seen that residual bullet will be deposited on the front of the target at the micro-scale. Furthermore, the *z*-stress distribution of whole model between MD and SPH at different moments under *u*_b_ = 6.0 km/s is presented in Figure 5b. The stress distribution calculated by these two methods are similar. Two compression waves are generated forward and backward at the impact surface when the bullet impacts the target. When these two compression waves reach the left surface of the bullet and the right surface of the target respectively, two rarefaction waves are generated that propagate into the model. Subsequently, the interaction of these two rarefaction waves leads to a tensile region. If this tensile stress is high enough, the spalled fragment shells on the surface of the bullet will occur. We then select the most severely deformed particle in the contact area (the front-side of the bullet) as the feature point for analysis. Comparison of the velocity and z-stress variation with time of feature particle between MD and SPH is in Figure 5c. The velocity/stress amplitude and evolution trend of the feature particle under these two methods are similar. It should be pointed out that the calculation results of velocity and stress have some inevitable errors affected by local deformation and non-equilibrium effects.

**Figure 6:**
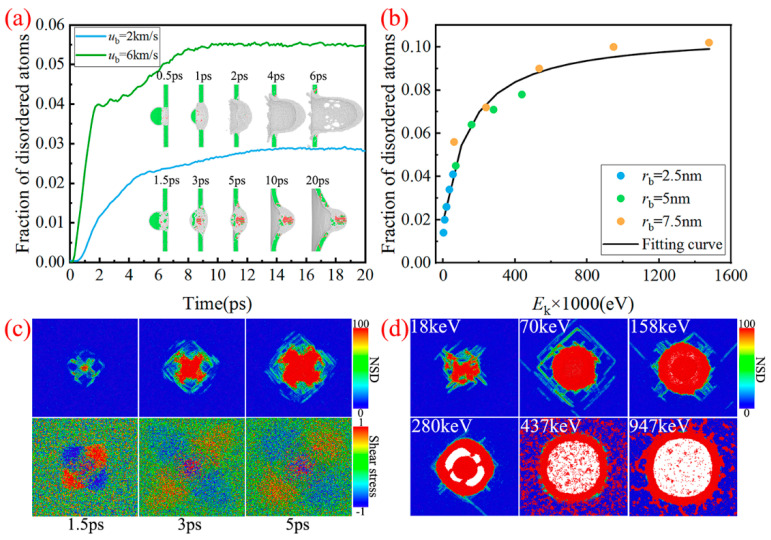
(**a**) The number of disordered atoms and the corresponding microstructure evolution. (**b**) Fraction of the disordered atoms in final state versus incident kinetic energy. (**c**) Evolution of nonaffine squared displacement (NSD) and shear stress with time (*u*_b_ = 2 km/s). (**d**) Nonaffine squared displacement caused by different initial kinetic energy. Atoms are color-coded by their local lattice structure [FCC (green), HCP (red) and disordered (white)] as obtained from dislocation extraction algorithm (DXA).

As we all know, the metallic strength depends on the creation and movement of dislocations within the crystal [42]. Generally speaking, the lower dislocation density and the greater obstacles to the dislocation movement render the higher material strength. In particular, the perfect metal crystals exhibit theoretical strength (~GPa), as the dislocation density is zero. However, a large number of dislocations (~10^10^ m^−2^) will be introduced into the metal during the actual preparation process, making its strength (~MPa) far less than the theoretical strength. Here, we count the number of disordered atoms, which shows strong plastic flow and even local melting. The corresponding microstructure evolution is displayed in Figure 6a. It can be seen that the number of disordered atoms increases rapidly from 0, and then gradually stabilizes due to the end of the penetration process. When the shock velocity is 2 km/s, the dislocations create in the contact area. As the penetration depth increases, the dislocations continue to extend outward. When the shock velocity increases to 6 km/s, the local melting occurs in the contact area between the bullet and the target. The dislocation atoms are significantly reduced, while the disordered atoms increase correspondingly. Figure 6b shows the fraction of disordered atoms in the final state versus incident kinetic energy. We can see that the number of disordered atoms continues to increase with the increasing incident kinetic energy. However, its growth rate gradually keeps decreasing. As can be seen, the fraction of disordered atoms increases exponentially with the increasing incident kinetic energy. The formalism of fitting line is given as follows:

(8) $$F_{disordered}=\exp\left(-2.24-\frac{111}{E_{k}+64}\right)\;$$

To better describe the melting process induced by the rising kinetic energy upon bullet impact, the evolution of nonaffine squared displacement (NSD) and shear stress with time are displayed in Figure 6c,d. Studies have shown that the decrease of local shear modulus is caused by the rise of nonaffine displacement, which leads to the decreasing material stiffness [43,44,45]. It can be seen from Figure 6c that the increase of the disordered atoms apparently gives rise to the nonaffine squared displacements. Correspondingly, the local shear stress decreases, especially in the impacted area, which indicates that the local shear modulus is decreasing. In addition, as the rising kinetic energy upon bullet impact, the nonaffine squared displacements gradually increase, resulting in the more serious local melting of the material (Figure 6d).

### 4.2. Comparison of Debris Cloud Characteristics

**Figure 7:**
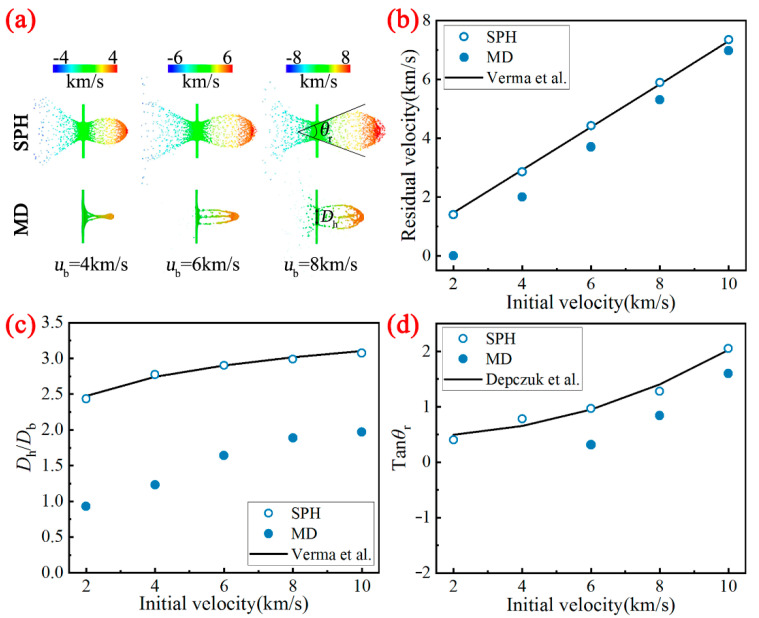
Comparison of (**a**) the morphology of debris clouds, (**b**) residual velocity, (**c**) crater diameter and (**d**) expansion angle between MD and SPH at different initial velocities.

The comparison of the debris cloud morphology based on the MD method and the SPH method under different impact velocities is shown in Figure 7a. Here, we take *τ* = 1 into account. At this time, the debris cloud characteristics have formed stably. It can be seen from Figure 7a that the debris cloud characteristics are significantly different. However, as the impact velocity increases, this difference between these two methods is getting smaller. To quantitatively investigate the difference of the debris cloud characteristics, the comparison of residual velocity, crater diameter and expansion angle between MD and SPH at different initial velocities is displayed in Figure 7b–d, respectively. Based on the empirical models developed by the researchers over a period of time and the generalized relation taking the following form of equation are found [1,46]

(9) $$\begin{array}{c} \frac{v_{r}}{v_{b}}=C_{1}\cdot {\left(\frac{u_{b}}{c}\right)}^{p1}\cdot {\left(\frac{D_{t}}{D_{b}}\right)}^{p2}\cdot \cos\theta +C_{2}\\ \frac{D_{h}}{D_{b}}=C_{3}\cdot {\left(\frac{u_{b}}{c}\right)}^{p3}\cdot {\left(\frac{D_{t}}{D_{b}}\right)}^{p4}\cdot \cos\theta +C_{4}\\ \tan\theta_{r}=C_{5}\cdot {\left(\frac{u_{b}}{c}\right)}^{p5}\cdot {\left(\frac{D_{t}}{D_{b}}\right)}^{p6}\cdot \cos\theta +C_{6} \end{array}$$

where *c* is the bulk sound velocity and *θ* denotes the angle between the flying velocity of the bullet and the normal of the target. We find that the Equation (9) is in good agreement with the SPH results when *p*1 = −0.36, *p*2 = 11.96, *p*3 = 0.027, *p*4 = −0.85, *p*5 = 2.24, *p*6 = −5.4, *C*_1_ = 2.88, *C*_2_ = 0.73, *C*_3_ = 5.46, *C*_4_ = −11.82, *C*_5_ = 1660, *C*_6_ = 0.45. More importantly, these three characteristics obtained by the MD method are all smaller than those obtained by the SPH method, especially for low-velocity conditions (the difference can be as much as two times). Note that under low impact strength (*u*_b_ = 2 and 4 km/s), the bullet will be embedded into the target, and cannot form a typical debris cloud morphology due to the high ductility of the material under the microscopic scale. Therefore, the corresponding expansion angles are not given here.

**Figure 8:**
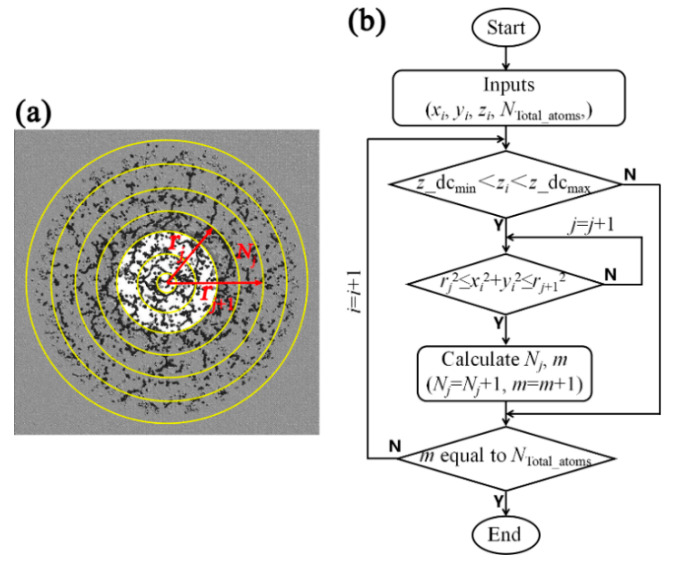
Schematic diagram (**a**) and flow chart (**b**) of the algorithm for solving the radial distribution of debris particles.

In order to calculate the radial distribution of the debris cloud, we take the *z*-axis as the center, and divide the entire debris cloud area into many concentric circles with the radius of *r*_j_. Thereby, many rings with the width of d*r* = *r*_j_ + _1_ − *r*_j_ are obtained, as shown in Figure 8a. Correspondingly, the flow chart of the algorithm for solving the radial distribution of debris particles is in Figure 8b.

**Figure 9:**
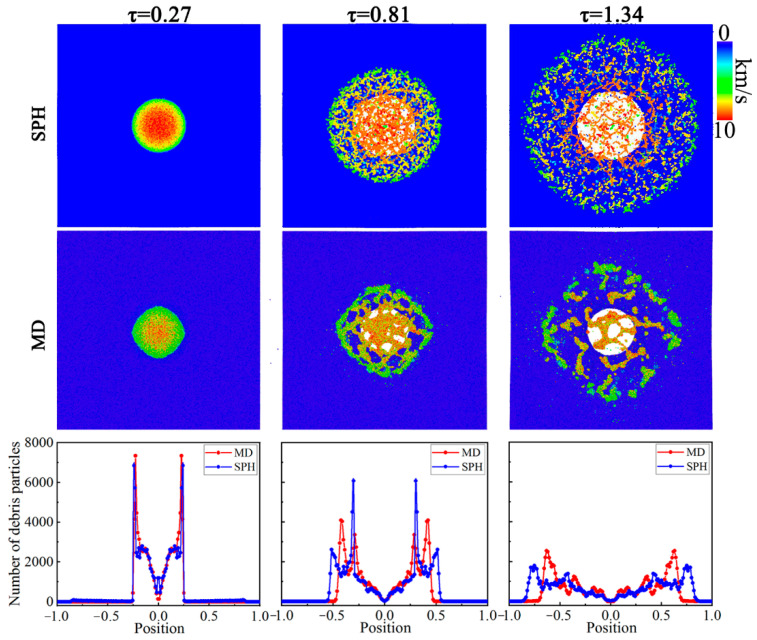
Comparison of the radial distribution of debris particles between MD and SPH at different moments under *u*_b_ = 10.0 km/s.

Based on the above division algorithm, we compared the distribution law of the MD method and SPH method at different moments, as shown in Figure 9. Here, we take *u*_b_ = 10.0 km/s into account. There is little difference in the radial distribution of debris clouds between the two methods when *τ* = 0.27. The debris particles are mainly located in the region of −0.25 ≤ *x*/*D*_t_ ≤ 0.25 (−0.25 ≤ *y*/*D*_t_ ≤ 0.25). Moreover, the number of particles gradually increases from the center of the debris cloud to the edge. As the debris cloud further expands (*τ* = 0.81), the peak number of particles decreases, while the width becomes wider (−0.5 ≤ *x*/*D*_t_ ≤ 0.5). It can be also seen that the radial distribution based on the SPH method is wider. When *τ* = 1.34, the debris cloud expands completely. Compared with MD method, the width of debris cloud under the SPH method is increased further, indicating that the debris cloud under this method expands faster.

## 5. Conclusions

This work investigates the difference in the fragmentation characteristics between macroscopic and microscopic results under a hypervelocity impact, with MD and SPH simulations. The content involves the debris cloud evolution, stress distribution, residual velocity, crater diameter and expansion angle. The main conclusions are summarized as follows:

(1) Compared with the typical microscopic debris cloud, the microscopic results show distinct differences. Under low shock intensity, the impacted area of the target gradually dents inward, and eventually protrudes on the back of target, showing good penetration resistance. Under high shock intensity, the width of the ejecta veil and external bubble of the debris cloud are narrower. More interestingly, the number of voids formed in the internal structure region is smaller, but the size is larger. In addition, the velocity decay rate and temperature rise rate of the bullet are much faster than those under the macro-scale.
(2) The propagation law of shock wave is very similar for the microscopic and macroscopic results. However, after the loading and unloading, the residual velocity of bullet, crater diameter and expansion angle of the debris cloud at the micro-scale are all smaller than those at the macro-scale, especially for low-velocity conditions. These characteristics indicate that the degree of conversion of kinetic energy to internal energy at the microscopic scale is much higher (by about one) than that of the macroscopic results.
(3) The MD simulation method can further provide more details of the physical characteristics at the micro-scale. Both the dislocation under low shock intensity and local melting under high shock intensity are shown. Furthermore, the number of disordered atoms increases rapidly from 0, and then gradually stabilizes due to the end of the penetration process. The fraction of disordered atoms then increases exponentially with the increasing incident kinetic energy.

## Figures and Tables

**Table 1:** Constitutive parameters of Al.

*ρ* (g/cm^3^)	*A* (MPa)	*B* (MPa)	Heat Capacity (J/(kg·°C))	Thermal Conductivity (w/(m·°C))
2.77	265	426	880	237

## Data Availability

The data presented in this study are available on request from the corresponding author.
